# Identification of Host Factors Differentially Induced by Clinically Diverse Strains of Tick-Borne Encephalitis Virus

**DOI:** 10.1128/jvi.00818-22

**Published:** 2022-09-13

**Authors:** Niluka Goonawardane, Laura Upstone, Mark Harris, Ian M. Jones

**Affiliations:** a School of Biological Sciences, University of Reading, Reading, United Kingdom; b School of Molecular and Cellular Biology, Faculty of Biological Sciences and Astbury Centre for Structural Molecular Biology, University of Leeds, Leeds, United Kingdom; University of North Carolina at Chapel Hill

**Keywords:** encephalitis, replicon, Spinach aptamer, luciferase, stress, interferon, TBEV, apoptosis, innate immunity, tropism

## Abstract

Tick-borne encephalitis virus (TBEV) is an important human arthropod-borne virus that causes tick-borne encephalitis (TBE) in humans. TBEV acutely infects the central nervous system (CNS), leading to neurological symptoms of various severity. No therapeutics are currently available for TBEV-associated disease. Virus strains of various pathogenicity have been described, although the basis of their diverse clinical outcome remains undefined. Work with infectious TBEV requires high-level biocontainment, meaning model systems that can recapitulate the virus life cycle are highly sought. Here, we report the generation of a self-replicating, noninfectious TBEV replicon used to study properties of high (Hypr) and low (Vs) pathogenic TBEV isolates. Using a Spinach2 RNA aptamer and luciferase reporter system, we perform the first direct comparison of Hypr and Vs in cell culture. Infectious wild-type (WT) viruses and chimeras of the nonstructural proteins 3 (NS3) and 5 (NS5) were investigated in parallel to validate the replicon data. We show that Hypr replicates to higher levels than Vs in mammalian cells, but not in arthropod cells, and that the basis of these differences map to the NS5 region, encoding the methyltransferase and RNA polymerase. For both Hypr and Vs strains, NS5 and the viral genome localized to intracellular structures typical of positive-strand RNA viruses. Hypr was associated with significant activation of IRF-3, caspase-3, and caspase-8, while Vs activated Akt, affording protection against caspase-mediated apoptosis. Higher activation of stress-granule proteins TIAR and G3BPI were an additional early feature of Vs but not for Hypr. These findings highlight novel host cell responses driven by NS5 that may dictate the differential clinical characteristics of TBEV strains. This highlights the utility of the TBEV replicons for further virological characterization and antiviral drug screening.

**IMPORTANCE** Tick-borne encephalitis virus (TBEV) is an emerging virus of the flavivirus family that is spread by ticks and causes neurological disease of various severity. No specific therapeutic treatments are available for TBE, and control in areas of endemicity is limited to vaccination. The pathology of TBEV ranges from mild to fatal, depending on the virus genotype. Characterization of TBEV isolates is challenging due to the requirement for high-containment facilities. Here, we described the construction of novel TBEV replicons that permit a molecular comparison of TBEV isolates of high and low pathogenicity.

## INTRODUCTION

Tick-borne encephalitis virus (genus Flavivirus, family *Flaviviridae*) is the causative agent of tick-borne encephalitis (TBE) (1), an important arthropod-borne disease of the central nervous system (CNS) that is endemic in parts of Europe and Asia (2, 3). Now recognized as a reemerged human pathogen, TBEV has spread into new geographical areas, with an estimated 14,000 TBEV cases reported across 30 European and Asian countries annually (4 to 7). In 2019, TBEV was isolated from ticks in the East of England, highlighting potential emergence in the United Kingdom (8, 9). Three closely related groups of TBEV exist, namely, European (TBEV-Eur), Far Eastern (TBEV-FE), and Siberian (TBEV-Sib), although more recent sequence-based analysis suggests further distinct subdivisions (10). Increased sampling in recent decades has further expanded the TBEV landscape with strains isolated in Eastern Siberia designated “Baikalean” isolates (genotype 4 [TBEV-Bkl-1] and genotype 5 [TBEV-Bkl-2]) (11 to 13), encompassing strain 886-84 and related East Siberian isolates (11, 14). All TBEV strains show high similarity at the nucleotide level (~84%), but the clinical outcome of infection is variable, ranging from asymptomatic in ~80 to 98% of cases to encephalitis of various severity (2 to 4, 15). The basis of this diversity remains undefined. TBEV is maintained by Ixodes ticks, with the majority of human TBEV infections caused by bites from I. ricinus (European and Far Eastern strains) or I. persulcatus (TBEV-Sib strain) (15 to 17).

TBEV is an icosahedral enveloped (~50 nm) virus with a positive-sense RNA genome of ~11 kb flanked by 5′- and 3′-untranslated regions (UTRs) (18, 19). The capped viral RNA is translated into a single polyprotein precursor that is cleaved by cellular and viral proteases to yield three structural proteins (C, prM, and E) and seven nonstructural proteins (NS1, NS2A, NS2B, NS3, NS4A, NS4B, and NS5) (18, 20). The C-terminal domain of NS5 encodes the viral RNA-dependent RNA polymerase (RdRp) (20 to 22). In common with other positive-sense RNA viruses, TBEV induces rearrangements of host membranes to establish compartmentalized viral factories (23 to 25). These are postulated to protect viral RNAs from host defenses (25, 26). TBEV infection triggers innate immune signaling through its interaction with RIG-I/MDA5, which promotes IRF-3 translocation to the nucleus (27, 28). Specific antiviral response genes have been shown to suppress TBEV replication in cell culture, including TRIM79α in mouse cells (29) and TRIM5α (30) and viperin (30) in human cells. The chemokine RANTES is upregulated in response to TBEV infection in human neuronal tissue via IRF-3 activation, and has been linked to TBEV neuropathology (28). TBEV also induces caspase-3 dependent apoptosis (31), which can be delayed by the interferon antagonist function of NS5 (32 to 35) and NS4A-mediated inhibition of STAT signaling (36). These mechanisms (summarized in reference 37) likely contribute to the variable clinical pathology of different TBEV isolates.

TBEV strain Vasilchenko (Vs) was first isolated in the Novosibirsk region of Russia in 1969 (38), and infectious clones have been generated (39). Vs causes a subclinical infection *in vivo* and shows minimal cytopathic effect (CPE) in cell culture (39). In contrast, strain Hypr, isolated in 1953 from an infected Czechoslovakian child, exhibits extensive CPE in cell culture and significant neuro-invasiveness in mice (40, 41). Despite these differences, Vs and Hypr are ~96% homologous at the amino acid level, permitting gene exchange studies to identify loci related to their pathogenicity. In this regard, Khasnatinov et al. developed Vs/Hypr chimeras revealing that the nonviremic transmission (NVT) of TBEV among ticks cofeeding on mice (42) is mediated by the 5′ region of the genome encoding the structural E protein. In contrast, CPE was mapped to the 3′ region encoding the nonstructural proteins. The precise regions governing these effects were not investigated in detail (42), and the clinical variability of these TBEV isolates remains largely undefined.

Here, to address this knowledge gap we generated a novel series of Hypr and Vs replicons and chimeras in which the structural genes were replaced with a *cis-*acting RNA tag Spinach2 (43, 44) to directly visualize RNA and the kinetics of virus replication. Chimeras in which the NS3 or NS5 regions of the Hypr/Vs were exchanged were further generated for the comparison of replication characteristics in both mammalian and invertebrate cell lines. The induction of early host innate immune defense proteins, including IRF-3, caspase-3, caspase-8, phospho-Akt, TIAR, and G3BPI, were also measured between Vs/Hypr chimeras in the context of replicon RNA and in infectious virus systems. We herein reveal key viral and cellular determinants that may contribute to the variable virus pathology of Hypr and Vs strains.

## RESULTS

### Generation of the Spinach2 TBEV replicon system.

**FIG 1:**
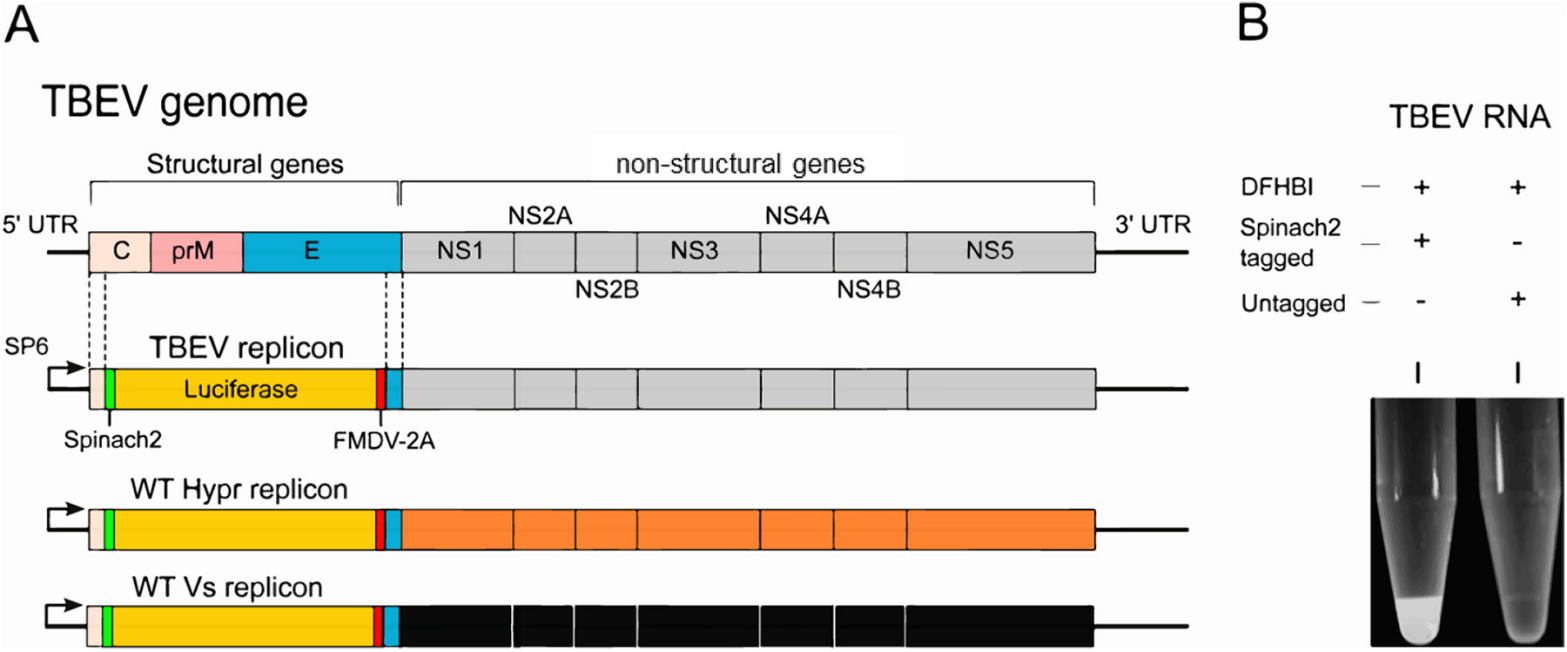
TBEV replicons. (A) Schematic representation of the TBEV genome (top) and replicon system in which the structural genes were replaced with Spinach2, FMDV-2A, and firefly (cu) luciferase. WT Hypr and Vs replicons are shown as a reference for subsequent chimeric construction. (B) Black-and-white image of RNA-fluorophore complexes from SP6-transcribed TBEV RNA (1 μg) mixed with the Spinach substrate DFHBI for 30 min.

Flavivirus replicons in which the structural genes are replaced with sequences encoding GFP or luciferase reporters (reviewed in reference 45), including for TBEV (46, 47), have been previously described. Upon transfection, both the markers and nonstructural proteins are translated, with the latter recognizing the 5′ and 3′ UTRs of the genome to establish active replication within cells. In this study, Hypr and Vs replicons were engineered to express a Spinach2 aptamer (43, 44), which forms an RNA sequence that folds to produce a fluorescent signal in the presence of 3,5-difluoro-4-hydroxybenzylidene imidazolinone (DFHBI). Replicons of Hypr and Vs were produced as surrogates for studies on infectious TBEV clones, as fully infectious virus systems are hazard group 3 viruses, requiring a high level of safety containment (BSL3) and rendering mutational/chimeric analysis unfeasible. Replicons were validated side-by-side with full-length infectious viruses to confirm their reliability for virological screening and assessment. The Spinach system has been used to visualize RNA replication in several systems (19, 48) but has not been previously reported for flaviviruses. The Spinach2 aptamer within a tRNA scaffold was incorporated into the +ve strand at the 5′ end of the UTR sequence, required for the formation of the secondary RNA structures (stem-loops 3 and 4) essential for genome cyclization sequence (Fig. 1A). Insertion of the Spinach2 RNA aptamer did not influence polyprotein synthesis. Firefly luciferase was cloned downstream of the Spinach2 sequence to provide a marker of TBEV translation. The firefly sequence was modified to contain no CpGs and a low UpA frequency (luc-cu) to enhance replication capacity as previously described (49). To ensure correct polyprotein processing and NS1 translocation to the ER (50), a ribosome-stuttering 2A peptide from foot-and-mouth disease virus (FMDV) was incorporated downstream of the luc-cu sequence prior to the transmembrane (TM) region of E (22 codons) (Fig. 1A). The ability of the replicon RNA to produce fluorescence was assessed following SP6-mediated transcription and incubation of the purified transcript with 10 μM DFHBI (Lucerna Technologies) and irradiation at 480 nm (Fig. 1B).

### Characterization of the Spinach TBEV replicon system.

**FIG 2:**
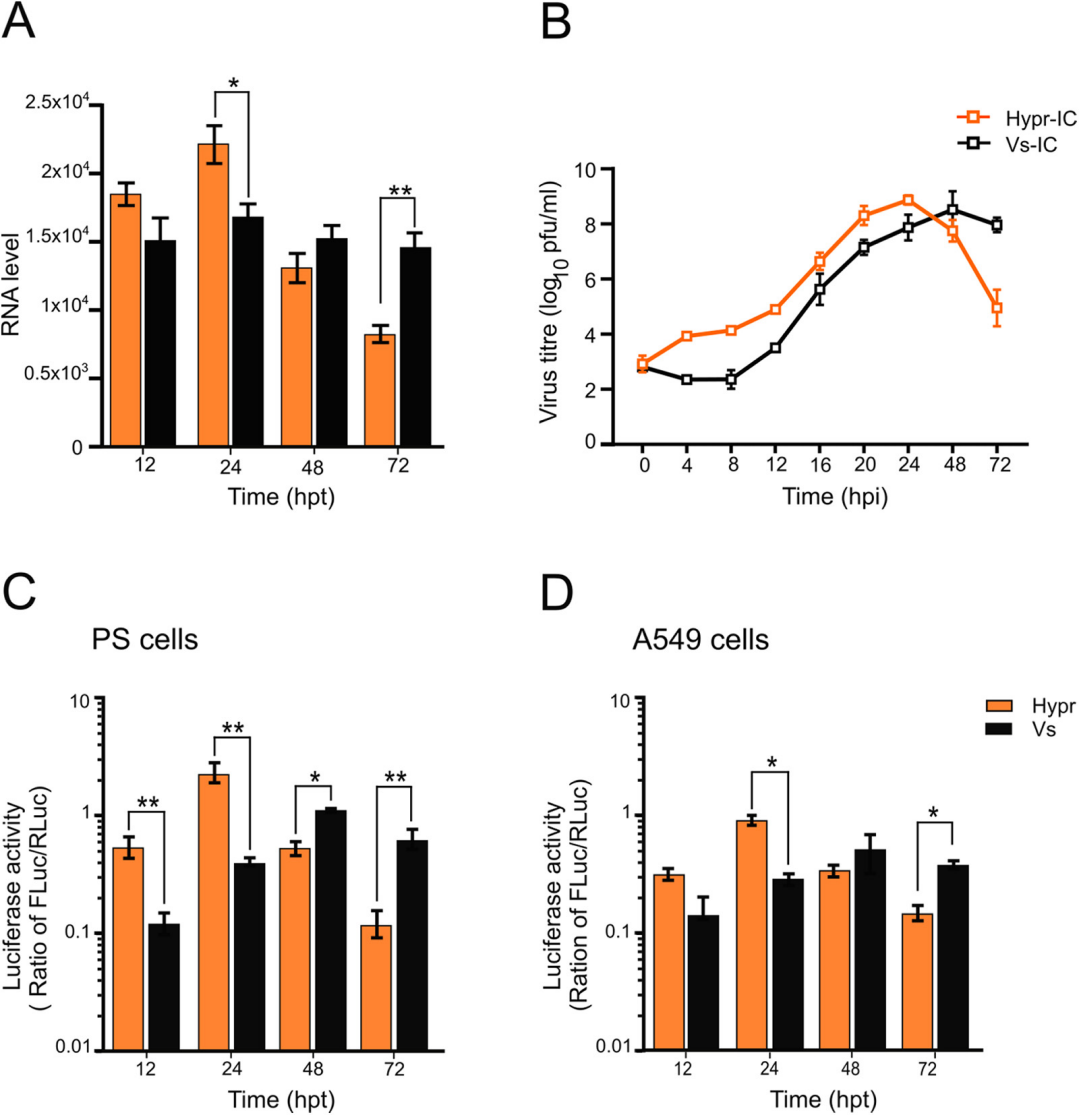
Characterization of the TBEV Spinach2 replicon in mammalian cells. (A) qRT-PCR analysis in TBEV replicon-transfected PS cells. Data are the mean ± SEM of two independent experiments. (B) Culture media collected from TBEV-infected PS cells (MOI 0.1) at the indicated times postinfection (pi) were assessed for virus infectivity via plaque assay. Data are the mean ± SEM of three independent experiments. (C and D) Luciferase assays of PS or A549 cells cotransfected with WT TBEV replicon (Hypr or Vs) and pTK-Ren (renilla) performed at the indicated time points posttransfection. Firefly values were normalized to renilla. Bar heights represent the mean ± SEM of three biological replicates. Assays were performed in triplicate. *, *P* < 0.01; **, *P* < 0.001, from WT Hypr determined using a two-tailed Student's *t* test with Welch’s correction.

To validate each genome assembly as a functional replicon, capped RNAs were transfected into porcine embryo kidney (PS) cells, previously shown to support active TBEV replication (15, 42, 51). Cells were harvested at defined time points posttransfection, and viral genomes were quantified using qRT-PCR (41). Upon analysis, both Hypr and Vs replicons showed evidence of genomic RNA synthesis from 12 hours posttransfection (hpt) (Fig. 2A). Upon assessment of the kinetics of viral RNA synthesis, the Hypr replicon showed robust replicative activity at early time points, peaking at 24 hpt and declining thereafter (Fig. 2A, orange bars). In contrast, the Vs replicon produced lower and more gradual levels of RNA synthesis that persisted over a longer time period than the Hypr replicon (Fig. 2A, black bars). Importantly, these features of Hypr and Vs were not restricted to the replicon system and could be confirmed in fully infectious virus systems using capped, *in vitro* transcribed WT virus RNA transfected into PS cells. Virus production was assessed by plaque assay of the cell supernatants at 24 h postinfection (hpi) (Fig. 2B), with Hypr showing more rapid virus production up to 24 hpi compared to the slower more sustained growth of Vs, validating the kinetics of the Spinach TBEV replicon system.

To ensure completion of the replication cycle in the Spinach2 TBEV replication system, firefly luciferase activity translated from progeny positive sense replicon RNAs, was measured in both PS (Fig. 2C) and A549 (Fig. 2D) cells. The latter are alveolar basal epithelial cells with a strong innate immune response, included as a representative human cell that is known to support flavivirus replication (25, 52). Replicon-derived luciferase activity, when normalized to the levels of cotransfected renilla luciferase, reflected the levels of +ve sense RNA, with an early but short-lived peak for Hypr and a slower, more prolonged signal for Vs (Fig. 2C and D). Of note, the rates of replication for Hypr and Vs were comparable between PS and A549 cells, suggestive of a conserved replicative phenotype across mammalian cell lines.

### Immunofluorescent visualization of the Spinach2 TBEV replicon system.

**FIG 3:**
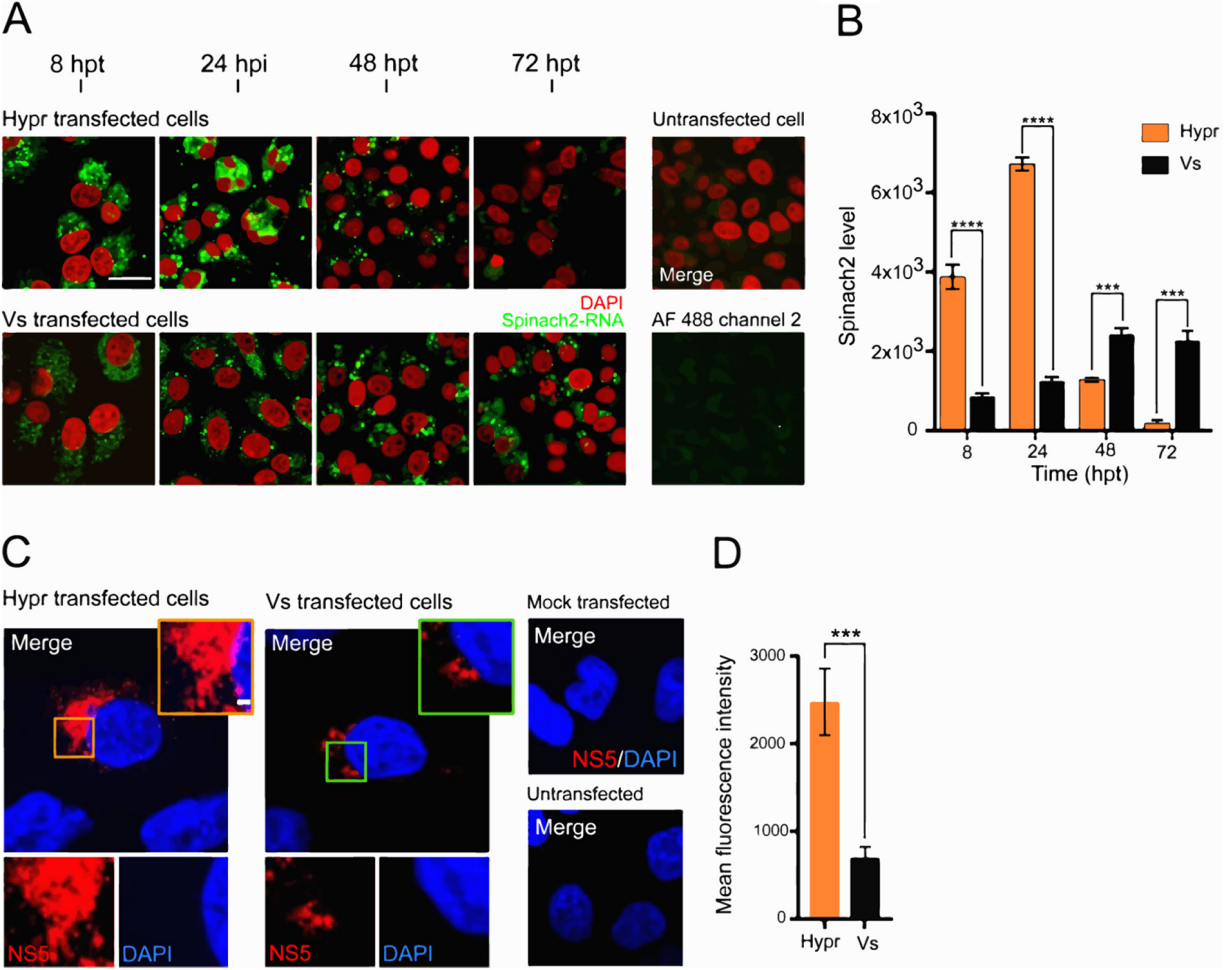
TBEV Spinach2 fluorescence in mammalian cells. (A) Time-course of DFHBI fluorescence (green) in PS cells transfected with Spinach2-tagged Hypr and Vs replicons. Nuclei were stained with Hoechst 33342 (red). Scale bar: 50 μm. (B) Quantification of Spinach2 replicon RNA performed using Fiji Image J (≥10 cells). Bar heights are the mean ± SEM of two biological replicates: ***, *P* <0.0003; ****, *P* < 0.0001 from WT Hypr. (C) Subcellular distribution of TBEV NS5 in PS cells transfected with *in vitro* transcribed Hypr and Vs replicons. At 24 hpt, cells were probed using an antiserum specific for TBEV NS5 and stained with goat rabbit IgG (H+L) conjugated to Alexa Fluor 488 (red). Nuclei were stained with DAPI (blue). Scale bar: 20 μm. (D) Quantification of NS5 expression using Fiji Image J (≤10 cells). Bar heights are the mean ± SEM of two biological replicates **, *P* < 0.001; ***, *P* < 0.0004, from WT Hypr determined using a two-tailed Student's *t* test with Welch’s correction.

To directly visualize replicating TBEV RNA, cells were incubated with DFHBI at defined time points posttransfection. Confocal analysis showed that the fluorescence of the Spinach labeled RNA was abundant in PS cells transfected with either Hypr or Vs Spinach2 replicons, but cells transfected with Hypr replicons were characterized by a granular and punctate staining pattern, which peaked at 24 hpt and substantially declined over the next 48 h (Fig. 3A, upper panel). In contrast, Vs replicons showed more diffuse fluorescence, only coalescing into a visible punctate pattern after 48 hpt, which then persisted for the duration of the analysis (Fig. 3A, lower panel). For both Hypr and Vs replicons, the predominant pattern of Spinach fluorescence was perinuclear, most notably at later time points, consistent with the formation of the virus-specific vesicles or spherules, as frequently observed for positive-sense RNA viruses (23 to 26, 53, 54). Quantitation of Spinach fluorescence was consistent with the observations: spike of Hypr fluorescence followed by a rapid decline, compared to gradual and more sustained increase detected for Vs replicon (Fig. 3B). Replicon-expressing cells stained with a rabbit NS5 antisera showed similar perinuclear aggregation, consistent with the sites of viral replication factories (Fig. 3C). The NS5 signal at 24 hpt was significantly greater for Hypr than Vs (Fig. 3D). These data confirm that Hypr and Vs show variable levels of replication in PS and A549 cells, with a pattern that broadly recapitulates the growth of each virus in mammalian cell culture (42).

### Differential replication of Hypr and Vs strains in mammalian cells is mediated by NS5.

**FIG 4:**
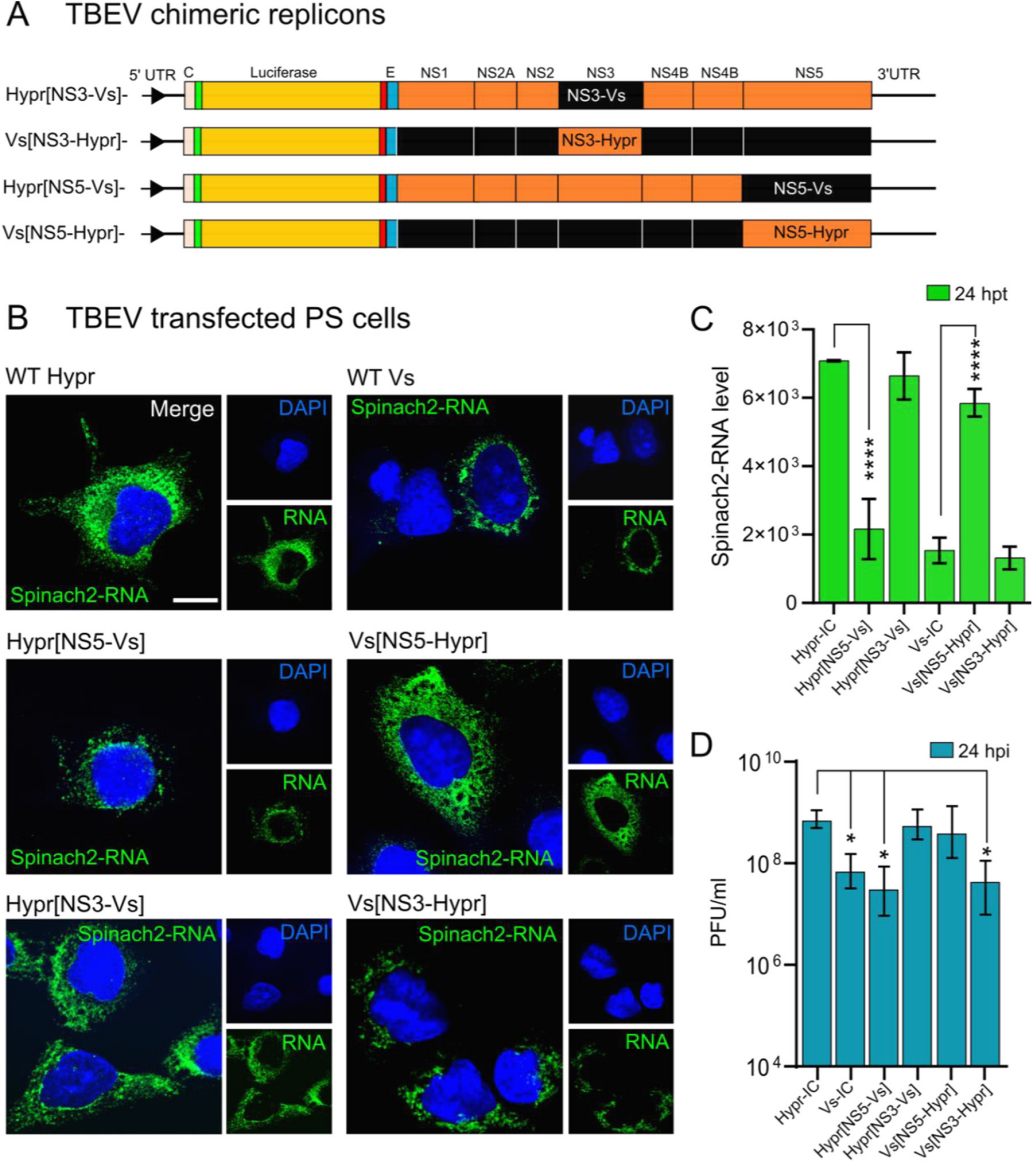
Subcellular localization of WT and NS5 and NS3 chimeric TBEV replicons and viruses in PS cells. (A) Schematic representation of TBEV NS3 (Hypr[NS3-Vs], Vs[NS3-Hypr]), NS5 [Vs(NS5-Hypr) and Vs(NS3-Hypr)]) chimeras. (B) Replicon transfected cells were treated with DFHBI at 24 hpt and fixed in 4% PFA. Cells were imaged on an Airyscan microscope. Nuclei were stained with DAPI (blue). Scale bar: 10 μm. (C) Quantification of Spinach2-RNA expression of WT versus chimeric replicons in PS cells. Bar heights are the mean ± SD of two biological replicates. ****, *P* < 0.0001 from WT. ns, no significant difference determined using a one-way ANOVA. (D) Quantification of plaque assays from PS cell supernatants transfected with the indicated TBEV replicons for 24 h. *, *P* < 0.01 compared to WT Hypr-IC determined using a one-way ANOVA.

The close relatedness of Hypr and Vs offers the opportunity to map the basis of their differential replication patterns through the creation of genetic chimeras between Hypr and Vs genomes. In the TBEV replicons described here, the lack of the structural region infers that differences between Hypr and Vs map to the NS coding region, previously shown to dictate virus-mediated CPE in cell culture (42). To further define these differences, chimeric replicons were created through the seamless exchange of Hypr and Vs sequences at the junction of NS3 or NS5, herein termed Hypr[NS3-Vs], Hypr[NS5-Vs], Vs[NS3-Hypr], and Vs[NS5-Hypr] (Fig. 4A). When PS cells were transfected with each chimeric replicon and assayed for Spinach fluorescence, early fluorescence signals associated with the Hypr replicon were lost upon exchange of the Hypr NS5 region with that of Vs. Conversely, the weaker fluorescence associated with the parental Vs replicon substantially increased upon replacement of Vs-NS5 with that of Hypr. In contrast, exchange of the NS3 coding region showed only a modest effect (Fig. 4B), with measurements at 24 hpt showing that the fluorescence associated with Vs Hypr-NS5 was as high as the parental Hypr strain, while that of Vs Hypr-NS3 showed no significant difference (Fig. 4C). Plaque assay of supernatants from cells infected with WT viruses (24 hpi) showed similar patterns: Vs with Hypr NS5 showed a 10-fold increase in infectivity, while Hypr with Vs NS5 showed a significant attenuation (Fig. 4D). The replication phenotypes observed for Hypr and Vs were similar to the levels of CPE detected for the parental viruses, highlighting the importance of the NS regions in governing these effects.

**FIG 5:**
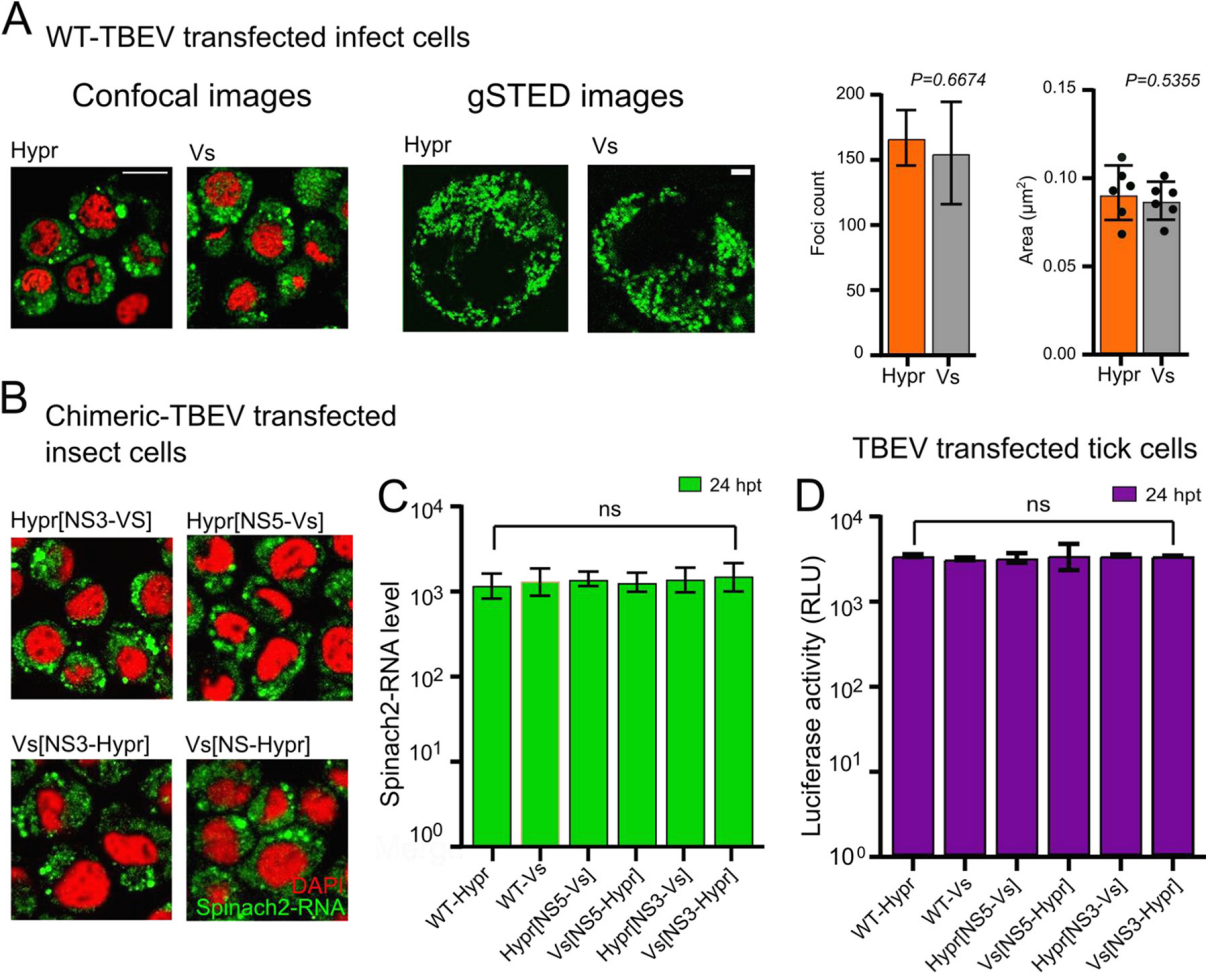
Replication of WT and NS5/NS3 chimeric replicons in invertebrate cells. (A) Sf9 cells were transfected with SP6 *in vitro* transcribed Spinach2 tagged (A) WT Hypr, Vs, or (B) chimeric replicons. (A) At 24 hpt, cells were incubated with DFHBI and live cells imaged on a Zeiss LSM780 confocal or Leica TCS SP8 gSTED microscope (upper left panel). Scale bars: 50 μm and 5 μm, respectively. Spatial data for gSTED-imaged Spinach2 RNAs determined using Fiji Image J (≥6 cells). Data were used to determine the Spinach2-RNA foci count per cell and average size per focus for Hypr and Vs (upper right graphs). (B) Spinach fluorescence from cells transfected with the indicated chimeric replicons (green). Cells were costained with DAPI (red). Scale bars: 50 μm. (C) Quantification of WT and chimeric Spinach2 RNA expression using Fiji Image J (≥10 cells for each chimera). (D) Luciferase assays of tick (IRE/CTVM19) cells cotransfected with TBEV replicons (WT or chimeras) at 24 hpt. Bar heights represent the mean ± SEM of three biological replicates. ns, no significant difference from WT Hypr determined using a one-way ANOVA.

TBEV is an arbovirus that replicates naturally in both tick and mammalian cells. To address if the differences observed in the replication kinetics of Hypr and Vs were conserved in invertebrate cells, Spodoptera frugiperda (Lepidoptera) cells were transfected with parental and chimeric replicons, and Spinach-associated fluorescence signals were assessed. In contrast to the phenotype in mammalian (PS and A549) cells, the Spinach-related fluorescence intensities of the parental Hypr and Vs replicons showed no obvious differences when imaged using standard confocal microscopy or gated stimulated emission depletion (gSTED) microscopy (Fig. 5A, left panels), with neither the number nor size of the fluorescent foci varying (Fig. 5A, right panels). Similar results were observed from NS3-NS5 chimeras (Fig. 5B). In all cases, the fluorescent signals were diffuse throughout the cells, which contrasted with the perinuclear distribution observed in PS cells. Quantification of the Spinach fluorescent signal at 24 hpt showed no significant differences among Hypr, Vs, and NS3–NS5 chimeric replicons, indicating comparable levels of replication (Fig. 5C). Similarly, all TBEV viruses showed comparable levels of replication in the tick cell line IRE/CTVM19 (Fig. 5D). These data suggest that the differences between Hypr and Vs in mammalian cells that maps to the NS5 region of the TBEV genome, is a product of the mammalian cell environment and not a product of the variable RdRp activity of the Hypr/Vs chimeras.

### Hypr and Vs differentially induce the innate immune response.

**FIG 6:**
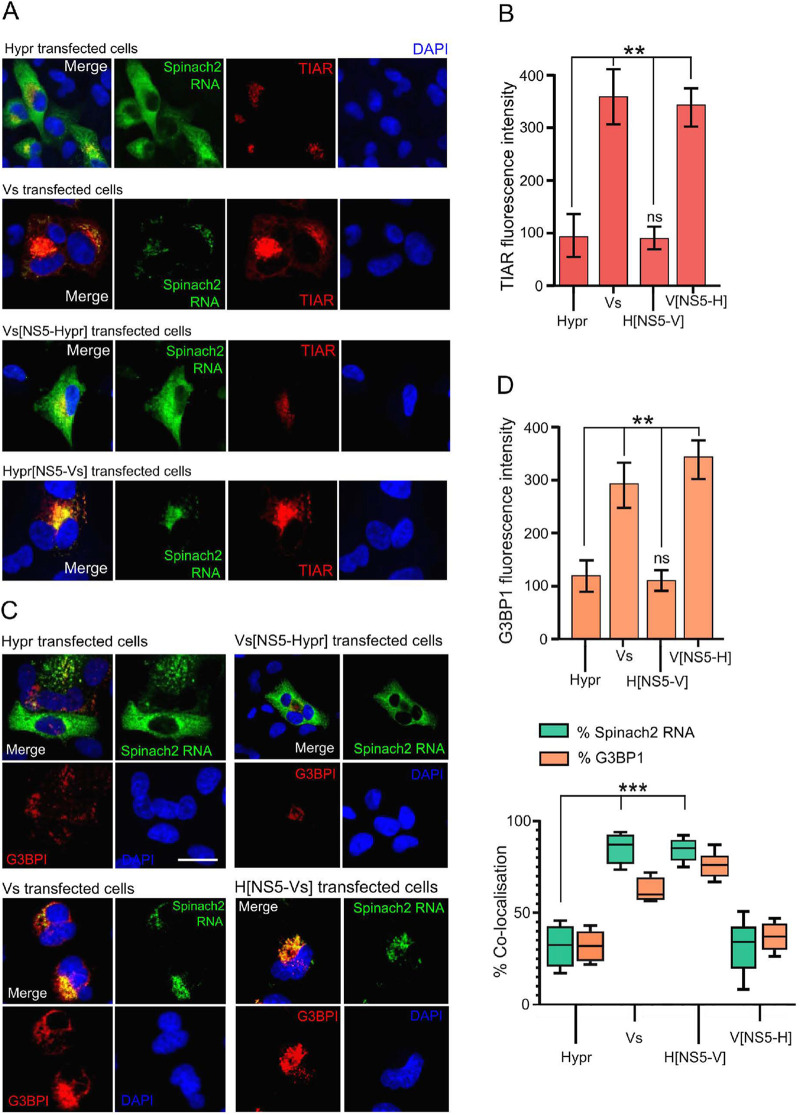
Formation of stress granules in TBEV replicon cells. (A) Replicon (WT or NS5 chimeras) and mock-transfected cells were fixed with 4% PFA at 24 hpt, incubated with DFHBI (Spinch2 signals indicated in green), and stained with anti-TIAR (red) and DAPI (blue). Cells were imaged on a Zeiss LSM780 confocal microscope. (B) Quantification of TIAR levels in WT Hypr- and-Vs or NS5 chimera transfected cells. Bar heights represent the mean ± SEM of two biological replicates. (C) Indicated replicon transfected cells were incubated with DFHBI (Spinch2 signals indicated in green), stained with anti-G3BP1 (red) and DAPI (blue), and imaged on an Airyscan microscope. Scale bar: 20 μm (left panel). (D) Quantification of G3BPI levels in WT Hypr- and Vs- or NS5-chimera transfected cells (upper left graph). Bar heights are the mean ± SEM of two biological replicates. Quantification of the colocalization of Spinach2 RNA with G3BP1 (green) or G3BPI colocalized with Spinach2 RNA (red). Manders’ overlap colocalization coefficients were calculated from ≤ 10 cells using Fiji, from at least two independent experiments (lower left graph). **, *P* < 0.001; ***, *P* < 0.0001 from WT Hypr determined using a two-tailed Student's *t* test with Welch’s correction.

**FIG 7:**
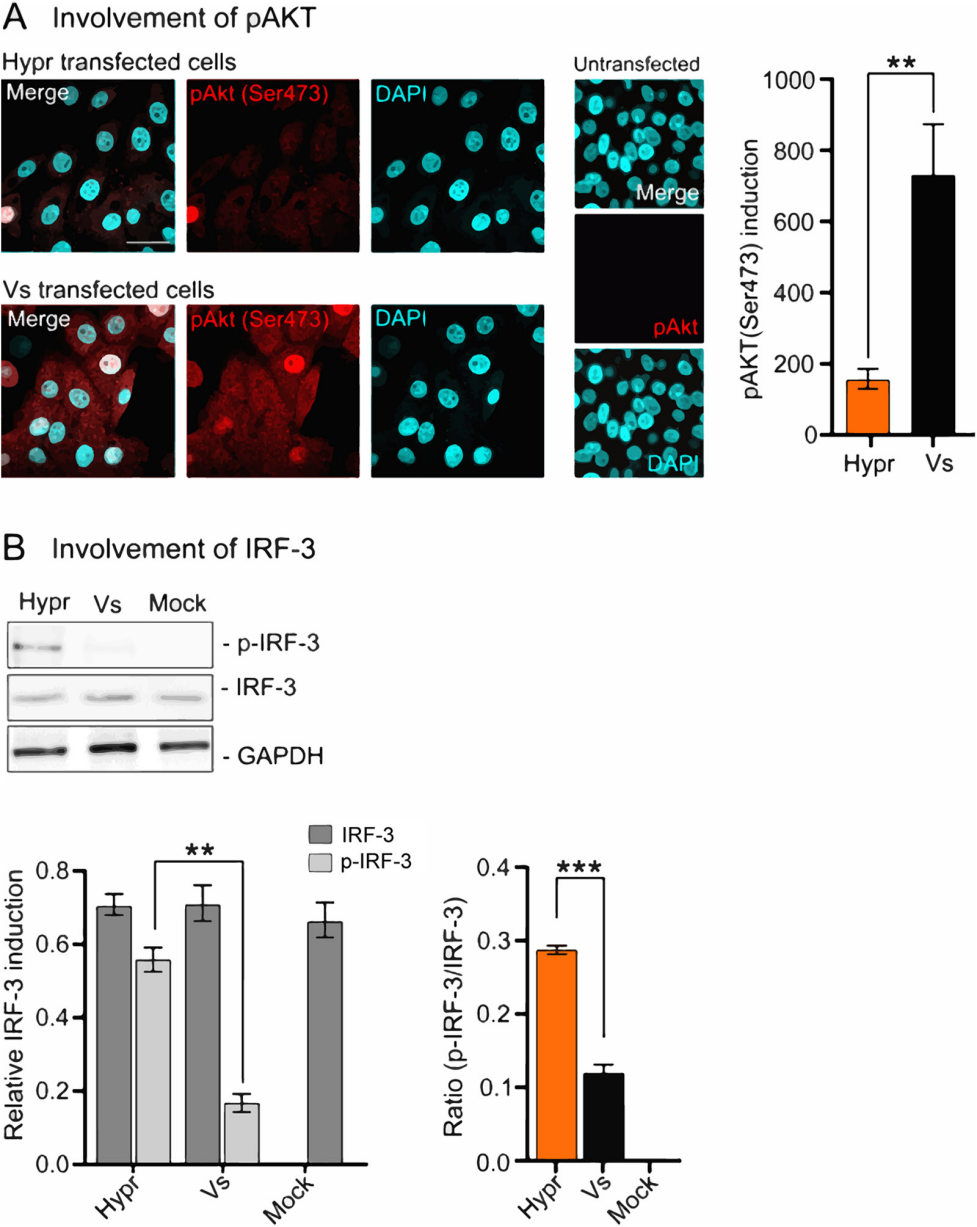
Levels of phosphorylated AKT and IRF-3 in TBEV replicon cells. (A) Immunofluorescence analysis of pAKT (Ser473) in Hypr- and Vs-transfected PS cells at 24 hpt. Cells were fixed with 4% PFA and stained with anti-pAKT (Ser473; red) and DAPI (cyan) and imaged on a Zeiss LSM780 confocal microscope. Scale bar: 50 μm (left panel). Quantification of pAKT (Ser473) in Hypr and Vs replicon transfected cells (right panel). Bar heights are the mean ± SEM of two biological repeats. (B) IRF-3 status in replicon transfected cells at 20 hpt analyzed by Western blotting for anti-pIRF-3 (Ser396) and anti-IRF-3. Lower panels represent the quantification of p-IRF-3 (Ser396), total IRF-3, and the ratio of p-IRF-3 to total IRF-3. Relative expression was normalized to GAPDH. Bar heights represent the mean ± SEM of three biological replicates. **, *P* < 0.0095; ***, *P* < 0.0001, from WT Hypr determined using a two-tailed Student's *t* test with Welch’s correction.

**FIG 8:**
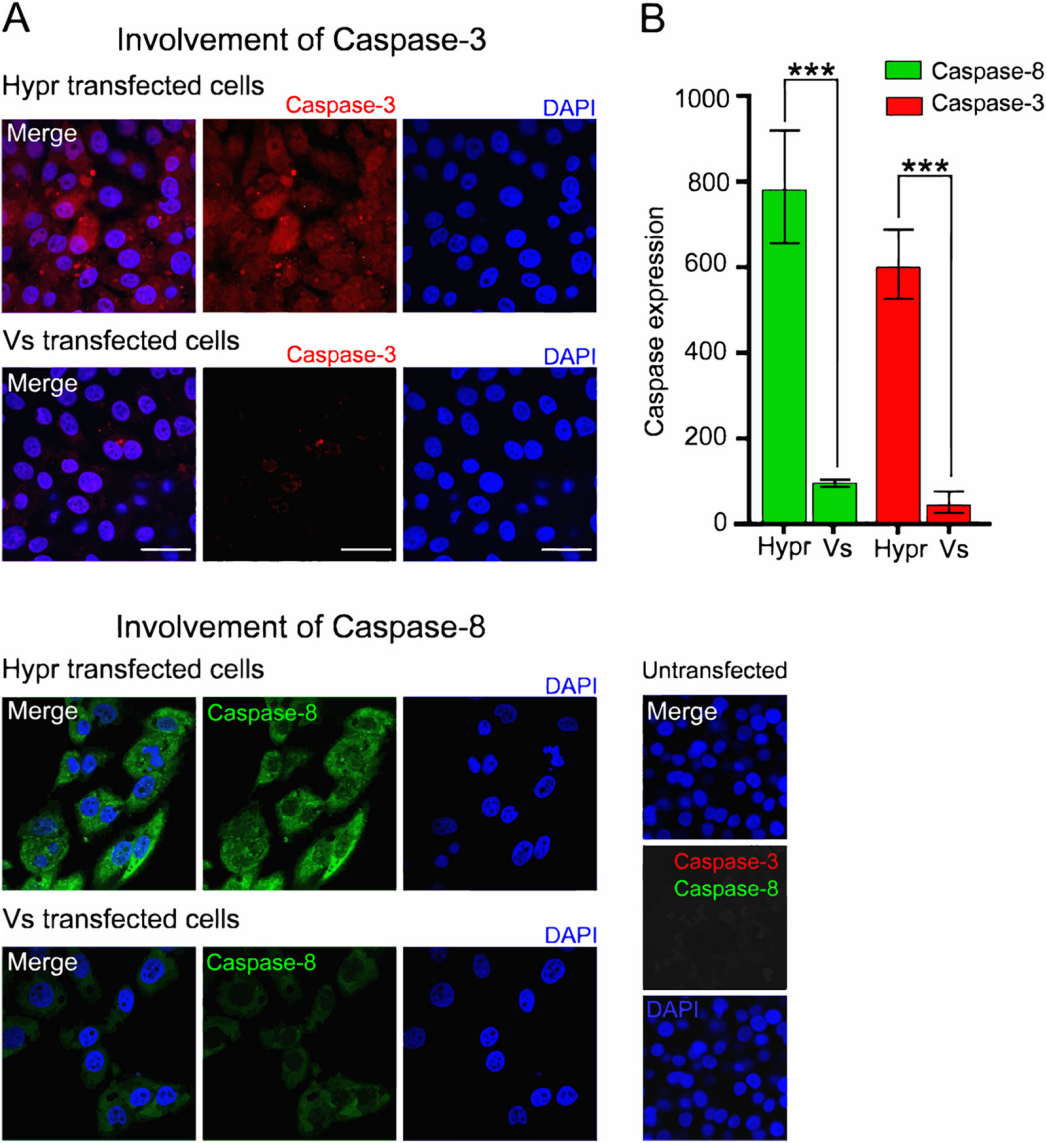
Hypr and Vs TBEV strains differentially induce caspases 3 and 8. (A) Immunofluorescence analysis of caspase-3 and -8 in Hypr and Vs transfected PS cells at 16 hpt. Cells were fixed with 4% PFA, stained with anti-caspase-3 (red), anti-caspase-8 (green), and DAPI (blue) and imaged on a Zeiss LSM780 confocal microscope. Scale bar: 50 μm. (B) Quantification of caspase-3 and -8 staining determined using Fiji Image J. Bar heights represent the mean ± SEM of three biological replicates. ***, *P* < 0.0001 from WT Hypr determined using a two-tailed Student's *t* test with Welch’s correction.

In invertebrate cells (tick or Sf9 cells), the interaction of TBEV with the innate immune system dictates the outcome of infection. The major mechanism(s) of antiviral defenses include siRNA systems that responds to viral RNAs to suppress viral replication through Dicer and Argonaut pathways (55). In mammalian cells, the detection of viral RNAs by sensors of the innate immune system trigger a signaling cascade that results in the induction of the interferon response and in some instances cell death (25, 51). To investigate whether immune sensing mediates the differences observed between Hypr and Vs strains in mammalian cells, the expression of key host defense markers were compared between cells expressing Hypr and Vs replicons. These included TIAR, TIA-1, and G3BP1, markers of stress granule formation that have previously been shown to bind to the TBEV genome (56, 57); phosphorylated AKT, a prosurvival kinase activated in TBEV-infected cells (58, 59); IRF-3, the canonical interferon response factor previously shown to be induced by TBEV NS5 (28); and caspase-8 and -3, inducer and executioner caspases of the apoptosis cascade (25, 60, 61), respectively. Upon analysis, stress granule (SG) formation was more extensive in Vs compared to Hypr replicon cells (Fig. 6, red panels). In Vs replicon cells, high levels of TIAR were observed in regions positive for viral RNA, compared to WT Hypr or Vs with Hypr-NS5 (Fig. 6A). Similarly, Hypr replicons with Vs-NS5 showed high levels of TIAR reminiscent of Vs (Fig. 6A, two lower panels, and Fig. 6B). Upon costaining for G3BP1 and Spinach2 (Fig. 6C, and Fig. 6D, top graph), the percentage of Spinach fluorescence colocalizing with G3BP1 in Vs- and Hypr[NS5-Vs]- transfected cells were significantly higher than Hypr/Vs[NS5-Hypr] (Fig. 6D, lower graph). Similarly, the levels of phosphorylated AKT (serine 473) in Vs replicon transfected cells were 5-fold higher than was observed in Hypr transfected cells (Fig. 7A). Induction of the interferon response measured through the levels of phosphorylated (serine 396) and nonphosphorylated IRF-3 showed a reciprocal pattern, with only low levels of pIRF-3 observed in Vs, compared to Hypr cells (Fig. 7B). Differential caspase activity between the two TBEV strains was also observed; Hypr replicons showed high levels of caspase-3 (cleaved at Asp175), which plays a central role in the execution phase of cell apoptosis, and caspase-8 (cleaved at Asp374), compared to the low levels observed for Vs (Fig. 8A and B). Hypr and Vs[NS5-Hypr] also showed a high abundance of caspase-3 and -8 and IRF-3 in PS cells when quantified by qPCR compared to Vs and Hypr[NS5-Vs] cells, which showed a high level of SG-related protein G3BP1 (Fig. S2 in the supplemental materials). These findings were consistent with the high levels of CPE observed for Hypr viruses and suggest that differential host cell defense responses to Hypr and Vs mediated by NS5 contribute to the replication characteristics observed for these viruses in mammalian cell culture systems.

## DISCUSSION

TBEV continues to spread throughout Europe, with emergence now reported in previously unaffected areas. In infected patients, specific TBEV strains lead to diverse clinical symptoms, ranging from mild to severe, a feature mirrored by their cytopathic effects (CPEs) in cell culture (42, 57, 62, 63). The mechanisms underlying the variability of these clinical symptoms remain unknown but are likely mediated by characteristics of the host and specific features of the TBEV genome (7, 42, 64). Studies with TBEV, however, require high containment facilities, making it challenging to perform experiments in fully infectious virus systems. In nature, TBEV strains with higher (Hypr) and lower (Vs) pathogenicity have been described. We therefore constructed noninfectious Spinach2-tagged replicons and chimeras of Hypr and Vs to investigate the basis of this differential pathogenicity. Previously designed TBEV replicons expressed eGFP or luciferase, which report only the final steady-state levels of viral protein expression. The Spinach-2 system is advantageous as it permits the direct measurement of viral RNA accumulation and localization in real time (43, 65).

We validated the TBEV replicons by assessing their ability to recapitulate virus replication kinetics (Fig. 2). We found that Hypr replicates to high levels at early time points, peaking at 24 hpt and declining thereafter (Fig. 2 and 4). This contrasted with the Vs replicon, which produces lower and more sustained levels of viral RNA synthesis that persist for longer (Fig. 2 and 4). These phenotypes were confirmed in Hypr and Vs viruses, validating the replicons for further chimeric assessments (Fig. 4D). Through construction of the Hypr/Vs chimeras (Fig. 4), we found that the key determinant of the differential replication kinetics maps to the NS5 protein. Exchange of the NS3 region, however, caused no discernible changes in Hypr or Vs replication. Of note, differences in Hypr and Vs were restricted to mammalian cell culture systems, with comparable replication kinetics observed among all Hypr and Vs replicons in invertebrate cells (Fig. 5). This strongly implicated the host cell response to infection as the key mediator of the differences between Hypr and Vs strains. This was further exemplified by differential levels of stress granule formation induced in mammalian cells with NS5 replicon chimeras of Hypr and Vs (Fig. 6).

NS5 possesses RNA cap methyltransferase (MTase) and RdRP activity, which mediates TBEV replication. Alignment of the ~911 amino acid sequences of Hypr and Vs NS5 show 47 changes, 15 of which lie within the N-terminal O-methyltransferase (O-MT) domain, while 32 map to the larger RdRp, with a small mutational bias toward O-MT (Fig. S1). NS5 has been ascribed a range of functions in TBEV-infected cells in addition to its direct role in virus replication, most notably its influence on Jak-STAT signaling and RANTES induction through the activation of IFN regulatory factor 3 (IRF-3) signaling that is dependent on RIG-I/MDA5 (28, 32, 36, 66). Of note, we observed several phenotypic differences in cells expressing Hypr and Vs Spinach2-replicons, including stress granule formation (Fig. 6), AKT and IRF-3 activation (Fig. 7), and caspase-8 and -3 induction (Fig. 8). These antiviral innate immune responses observed for Vs replicons prevent translation and arrest viral gene expression (56, 57), which may explain the reduced levels of replication compared to Hypr. The abundance of caspase-3,-8 stress granules (G3BP1) and IRF-3 were confirmed at the mRNA level (Fig. S2) and were also predominantly regulated by NS5. While the precise mechanisms by which TBEV viruses regulate host stress granules are yet to be confirmed, Hypr-NS5-induced IRF-3 expression would be predicted to drive innate immune defenses and subsequent neuroinflammation, a common feature of pathogenic TBEV viruses. In support of this, recent studies highlight how cell type-specific innate immunity contributes to shaping TBEV tropism and ultimately disease outcome in human brain cells (67, 68).

By extension, differential innate responses might be critical to the clinical outcome of flaviviruses (2, 31, 63). This is exemplified by vesicular stomatitis virus, which can switch tropism from peripheral to the central nervous system (CNS) based on the strength of the interferon (IFN) response in the lymph node subcapsular region, which acts as a gateway between the vasculature and the CNS (69, 70). Minor changes in innate immune antagonism due to virus emergence from the quasi-species may therefore account for the apparent gross clinical differences observed across TBEV strains. It is notable that differential triggering of innate sensors such as RIG-I also underlie the variable cytokine induction observed by different influenza virus strains with variable clinical outcomes (71).

No specific treatments for TBEV infections are currently available, and control is achieved by a prophylactic vaccine offered in areas of endemicity and for those who travel to them (5, 6, 72). Our data suggest that interventions to moderate the activities of NS5 may reduce the virulence of TBEV through the suppression of its ability to modulate host defenses. The finding that the replication kinetics of specific TBEV strains can be easily monitored by the Spinach2-replicon system permits screening of a range of current and emerging TBEV strains as part of preparedness. The importance of key amino acid substitutions in pathogenic versus nonpathogenic TBEV with these replicons can also bypass studies with full-length infectious cDNA clones that require BSL3 containment, advancing our understanding of TBEV pathogenesis. A comprehensive assessment of these replicons in a range of neuronal and tick cell systems will form the basis of future studies that aim to fully define how NS5 and innate immune defenses control TBEV pathogenicity and the subsequent variability in disease presentation.

## MATERIALS AND METHODS

### Cells.

Porcine embryo kidney (PS) cells were grown in RPMI 1640 (Gibco) supplemented with 5% heat-inactivated fetal calf serum (FCS) at 37°C in a CO_2_ (5%) incubator. A549 cells were maintained in Dulbecco’s modified Eagle medium (DMEM) (Life Technologies) supplemented with 10% heat-inactivated FBS (Gibco). Unless otherwise stated, replicon transfected cells were maintained in media supplemented with 2% FCS and 1% Penicillin-Streptomycin (Invitrogen). Sf9 cells (ATCC) were cultured in BioWhittaker Insect-Xpress supplemented with 2% FCS, 1% Penicillin-Streptomycin, and 2.5 μg/mL amphotericin B. Cells were grown at 28°C as monolayers or in suspension with agitation at 100 rpm. Transfection of TBEV replicons were performed in Sf9 monolayer cultures. IRE/CTVM19 was maintained in ambient air at 28°C in L-15 (Leibovitz) medium supplemented with 10% tryptose phosphate broth (TPB), 20% FCS, 2 mM l-glutamine, and Penicillin-Streptomycin (55).

### Construction of TBEV replicons.

cDNA clones of WE-TBEV strain Hypr (U39292) or SIB-TBEV strain Vs (AF069066) (39, 42) were positioned downstream of the SP6 promoter, from which infectious RNA could be transcribed *in vitro.* Chimeric replicons were constructed through restriction fragment swapping, using intermediate plasmids as required, or through synthesis of DNA fragments *de novo* followed by Gibson assembly (73) using NEBuilder HiFi (NEB). All clones were fully sequenced for verification.

### Recovery of replicon RNA.

DNA plasmids encoding the relevant clone or chimeras were linearized at the SmaI restriction site downstream of the TBEV coding sequence and used as a template to produce full-length capped RNA using SP6 RNA polymerase (Promega). Each reaction (50 μL total volume) contained 1 μg of linearized DNA template and 40 units of SP6 RNA polymerase, incubated at 37°C for 3 h. RNAs were purified using the SV total RNA isolation kit (Promega) and resuspended in RNase-free water (Invitrogen). RNA integrity was verified by agarose gel (1%) electrophoresis and quantified by spectrophotometry.

### RNA transfections.

Transfections were performed using Lipofectamine 2000 (Invitrogen) as per the manufacturer’s recommendations. Briefly, 1 μg of SP6 transcribed RNA was complexed with the transfection reagent in serum- and antibiotic-free culture medium for 10 min at room temperature (RT). Complexes were added to cell monolayers for 24 to 96 h. All transfections were performed in triplicate.

### Plaque assay: titration of infectious TBEV.

Full-length TBEV RNAs were produced and purified as described above. PS cells were transfected with SP6-transcribed RNAs (1 μg RNA for 1.2 × 10^5^ cells) using Lipofectamine 2000 (Invitrogen) as per the manufacturer’s recommendation. Infectious supernatants were collected at 24 to 72 hpi. Aliquots of virus were diluted with serum-free RPMI 1640 and applied to monolayers of PS cells for 1 h at 37°C. The inoculum was aspirated, and plates were overlaid with RPMI 1640 supplemented with 2% FCS and 1% SeaPlaque Agarose (Cambrex) for 5 days at 37°C for plaque formation. Monolayers were fixed with 4% paraformaldehyde and stained with 0.05% crystal violet. Plaques were counted and virus titers expressed as log_10_ PFU/mL. All virus work was performed in a Biological Safety Level 3 (BSL3) laboratory. For TBEV growth kinetics, PS cells were infected with WT or NS3/NS5 chimeras at a multiplicity of infection (MOI) of 0.1 for 1 h. Infected cells were washed five times with PBS and incubated with fresh complete medium (2% FCS) at 37°C. Supernatant from infected cells was collected at 0, 4, 8, 12, 16, 20, 24, and 72 hpi and frozen at −80°C prior to further analysis. Experiments were performed in triplicate. Titers of infectious virus at different time points were determined by plaque assay.

### Immunofluorescence (IF) assay.

For IF analysis, transfected cells were cultured in 35-mm glass-bottomed dishes (MatTek Corp.), fixed with 4% paraformaldehyde (Applichem GmbH), and permeabilized in PBS-T (0.1% vol/vol Triton X-100 in PBS) for 5 min. Cells were blocked in PBS-T containing 5% wt/vol bovine serum albumin (BSA) for 10 min and probed with primary (Rabbit MAb) antibodies purchased from Cell Signaling Technology (CST) (1:100, cleaved caspase-8 [Asp391, 18C8], cleaved caspase-3 [Asp175, 5A1E], Phospho-Akt [Ser473, D9E] XP, and TIAR [D32D3] XP) or Sigma (1:500, G3BPI: G6046, RRID:AB_1840864) in PBS-T, 1% wt/vol BSA for 1 h at RT. Cells were washed in PBS and stained with 1:500 fluorochrome-conjugated secondary antibodies in PBS-T, 1% wt/vol BSA for 1 h at RT in the dark. Nuclei were counterstained with Hoechst 33342 DNA dye NucBlue Live ReadyProbes (Thermo Fisher) reagent for live cell imaging or 4’,6’-diamidino-2-phenylindole dihydrochloride (DAPI) and SYTO60 fluorescent nucleic acid stain for fixed samples (Invitrogen).

### Detection of Spinach2 tagged replicon RNA.

For live-cell analysis of Spinach2 expression, cells were imaged 24 to 96 h posttransfection. Cell cultured medium was replaced with imaging media (RPMI 1640 without phenol red or vitamins, supplemented with 25 mM HEPES, 5 mM MgSO4), 20 μM DFHBI (Lucerna technologies) 30 min prior to analysis. Images were analyzed using NIS-Elements software. Background intensities were subtracted from all pixel intensity measurements to avoid noise in the final images.

### Confocal microscopy.

Live fluorescence images were obtained on an A1R laser scanning microscope (LSCM) using an A1^+^ galvano scanner and oil immersion objective (Nikon). Spinach2 was imaged using an FITC 488 nm laser for EGPF (470/40 excitation and 515/30 emission). Nuclei were stained with NucBlue Live Ready Probes (Invitrogen) and detected using DAPI filters (emission filter 450/35 nm). NS5 protein and SGs images were obtained using a Leica TCS SP8 confocal microscope with a 592-nm and/or a 660-nm depletion laser and an HCX Plan Apo 100×/1.4 oil objective. TBEV chimeric images were acquired on a Zeiss LSM880 microscope with Airyscan. Postacquisition analysis was performed using Zen (Zen version 2015 black edition 2.3, Zeiss), Leica LAS X, or Fiji Image J (v.1.49) software (74).

### Image analysis.

Images were analyzed using the Fiji package of Image J. For quantification of the spatial distribution of Spinach2-RNA, images were acquired under identical parameters, but with a variable gain to ensure correct exposure. Two-dimensional areas and foci counts of aptamer-tagged RNAs were measured using the Analyze Particles function in Fiji. Briefly, RGB channels were split, resulting in grayscale images, which were threshold adjusted to convert to binary images. The Watershed function was used to separate overlapping objects based on their circularity. The Analyze Particles function was used to calculate the number of foci in a given image. For fluorescence intensity measurements, the binary image for single particle detection was used to create a mask, and the mean pixel intensity was calculated for each particle (*n* ≥ 10 cells from at least two independent experiments).

For colocalization analysis, Manders' overlap coefficients were calculated using Fiji software with Just Another Co-localization Plugin (JACoP) (National Institutes of Health) (75, 76), where the M1 coefficient reports the fraction of the Spinach2-RNA signal that overlaps with the anti-G3BP1 signal, while the M2 coefficient reports the fraction of the anti-G3BP1 signal that overlaps with the Spinach2-RNA signal. Coefficient values ranged from 0 to 1, corresponding to nonoverlapping images and 100% colocalization, respectively. Colocalization was assessed on ≥ 10 cells from at least two independent experiments.

### Luciferase assays.

Cells were seeded at 1.5 × 10^5^ cells per well in 96-well plates (*n* = 3) and transfected with *in vitro* transcribed TBEV replicon RNA (100 ng/well) and 10 ng of pTK-Ren (Promega) using Lipofectamine 2000 (Thermo Fisher). Cells were harvested in passive lysis buffer (Promega) at 12 to 72 hpt, and luciferase activity was measured using the Dual Luciferase reagent kit and GloMax multidetection system (Promega). Firefly luciferase readings from the TBEV replicons were normalized to the renilla values (pTK-Ren) of each sample.

### Western blotting.

IRF-3 and phospho-IRF-3 (Ser396) were monitored 20 h posttransfection of the TBEV replicon. PS cells (1.5 × 10^6^ cells/well in 6-well format) were harvested by centrifugation at 12,000 × *g* for 2 min, mixed with an equal volume of sample buffer (Bio-Rad) and boiled for 10 min. Samples were separated by polyacrylamide gel electrophoresis and transferred to nitrocellulose membranes (Bio-Rad). Membranes were blocked in 0.1% vol/vol Tween 20 in PBS (PBS-T) and 5% wt/vol nonfat milk at RT for 1 h. The following antibodies were probed: rabbit phospho-IRF-3 (Ser396) (4D4G) MAb (1:1,000, CST, 4947), rabbit IRF-3 (1:1000, CST, 4302), rabbit anti-GAPDH polyclonal antibodies (1:5,000, Proteintech, 10494-1-AP), and horseradish peroxidase (HRP)-conjugated anti-rabbit IgG polyclonal antibodies (CST). Chemiluminescence was detected using the ChemiDoc Touch Imaging System (Bio-Rad). Images were analyzed using Image J software. Quantitative data were obtained for two independent experiments.

### RT-PCR analysis.

RT-PCRs were performed using a one-step protocol in a total reaction volume of 25 μL. Primers and probes were as follows: forward-(5′-GGGCGGTTCTTGTTCTCC-3′), reverse-(5′-TGAGCCACCATCACCCAGACACA-3′), and probe (FAM-ACACATCACCTCCTTGTCAGACT-TAMRA) (41). The reaction mixture contained 1 μL of sample RNA, 12.5 μL 2× Reaction Mix, 0.5 μL SuperScript III Platinum One-Step Taq Mix (Invitrogen), 300 nM forward primer, 900 nM reverse primer, and 250 nM of the TBE-WT probe. Cycling conditions were as follows: 30 min reverse transcription at 42°C, denaturation for 10 min at 94°C, followed by 40 cycles for 15 sec at 95°C and 1 min at 60°C. qPCRs were performed using the StepOnePlus Real-Time PCR system (Applied Biosystems).

For the quantification of mRNA abundance, total cellular RNA was isolated using RNeasy kit (Qiagen). qRT-PCRs were performed on 50 ng of RNA using Superscript III (Invitrogen) and Fast SYBR Green Master Mix (Applied Biosystems) as per the manufacturer’s recommendations. Primers used for qPCRs are as follows: GAPDH: forward-(5′-GGAGCGAGATCCCTCCAAAAT-3′) and reverse-(5′-GGCTGTTGTCATACTTCTCATGG-3′); caspase-3: forward-(5′-CATGGAAGCGAATCAATGGACT-3′) and reverse-(5′-CTGTACCAGACCGAGATGTCA-3′); caspase-8: forward (5′-TTTCTGCCTACAGGGTCATGC-3′) and reverse-(5′-GCTGCTTCTCTCTTTGCTGAA-3′); G3BP1 forward-(5′-GAA ATC CAA GAG GAA AAG CC-3′) and reverse-(5′-CCC AAG AAA ATG TCC TCA AG-3′); IRF3: forward-(5′-ACC AGC CGT GGA CCA AGA G-3′) and reverse-(5′-TAC CAA GGC CCT GAG GCA C-3′). mRNA abundance was normalized to GAPDH to obtain ΔCt values. In each qPCR assay, a mean threshold cycle (Ct) was obtained from two independent samples performed in triplicate. Differences were normalized to reference samples (uninfected cell control) and calculated as a final ΔCt = ΔCt_reference_ – ΔCt_sample_. Relative transcript abundance was calculated using final ΔCt as 2^ΔCt^.

### Statistical analysis.

Statistical significance was determined in GraphPad Prism using a Student's *t* test with Welch’s correction or a one-way ANOVA with Bonferroni's correction.
