# Supplementary material for: Identification of Host Factors Differentially Induced by Clinically Diverse Strains of Tick-Borne Encephalitis Virus
Source: J Virol. 2022 Sep 13;96(18):e00818-22. doi: 10.1128/jvi.00818-22 (PMC9517736; doi:10.1128/jvi.00818-22)
Supplement: Supplemental file 1 — Fig. S1 and S2. Download jvi.00818-22-s0001.pdf, PDF file, 2.1 MB [file jvi.00818-22-s0001.pdf]

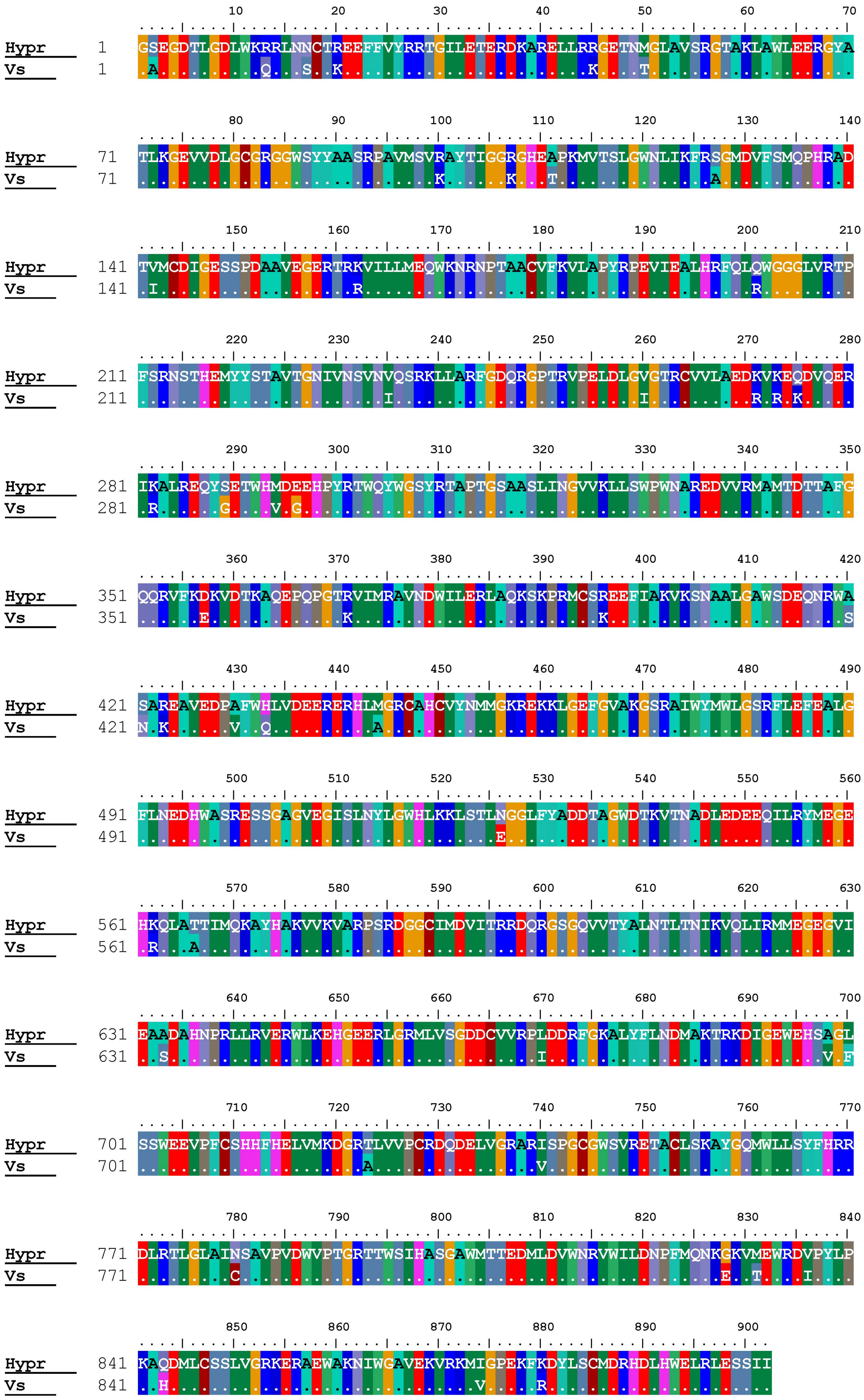

Fig S1. Sequence alignment of full-length NS5 in Hypr and Vasilchenko (Vs) strains. GenBank accession numbers: Hypr (U39292) and Vasilchenko (AF069066). Conserved residues are indicated by the dotted lines.

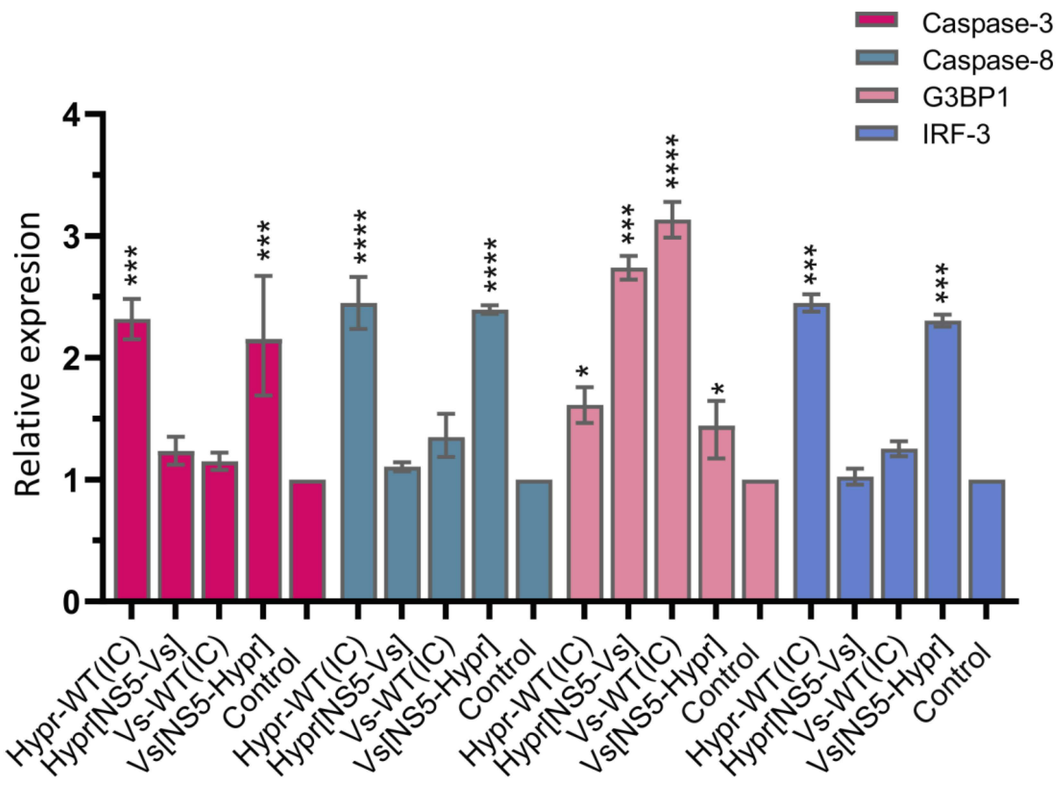

Fig S2. Regulation of mammalian host gene expression in Hypr/Vs or NS5 chimeras. PS cells were transfected with Hypr/Vs or the indicated NS5 chimeras (1 µg) for 24 h and mRNA levels of Caspse-3, -8, G3BP1 and IRF-3 were determined by SYBR green based quantitative PCR. GAPDH was included as an internal control. Data represent the mean SE from two independent experiments. Untransfected cells were compared as a control. ***, P=<0.0004 and ****, =<0.0001 **** from uninfected controls determined using a two-tailed Student's *t*-test with Welch's correction.
